# Novel ultrasound techniques in the identification of vulnerable plaques—an updated review of the literature

**DOI:** 10.3389/fcvm.2023.1069745

**Published:** 2023-05-24

**Authors:** Yujuan Yao, Pingyang Zhang

**Affiliations:** Department of Cardiovascular Ultrasound, Nanjing First Hospital, Nanjing Medical University, Nanjing, China

**Keywords:** contrast-enhanced ultrasound (CEUS), ultrasound molecular imaging (UMI), elastography, intravascular ultrasound imaging (IVUS), diagnosis, vulnerable plaque

## Abstract

Atherosclerosis is an inflammatory disease partly mediated by lipoproteins. The rupture of vulnerable atherosclerotic plaques and thrombosis are major contributors to the development of acute cardiovascular events. Despite various advances in the treatment of atherosclerosis, there has been no satisfaction in the prevention and assessment of atherosclerotic vascular disease. The identification and classification of vulnerable plaques at an early stage as well as research of new treatments remain a challenge and the ultimate goal in the management of atherosclerosis and cardiovascular disease. The specific morphological features of vulnerable plaques, including intraplaque hemorrhage, large lipid necrotic cores, thin fibrous caps, inflammation, and neovascularisation, make it possible to identify and characterize plaques with a variety of invasive and non-invasive imaging techniques. Notably, the development of novel ultrasound techniques has introduced the traditional assessment of plaque echogenicity and luminal stenosis to a deeper assessment of plaque composition and the molecular field. This review will discuss the advantages and limitations of five currently available ultrasound imaging modalities for assessing plaque vulnerability, based on the biological characteristics of the vulnerable plaque, and their value in terms of clinical diagnosis, prognosis, and treatment efficacy assessment.

## 1. Introduction

Atherosclerosis, characterized by the formation of lipid-rich plaques in the arterial wall, is the pathological basis of cardiovascular disease, which remains the major underlying factor of morbidity and mortality worldwide (1). Atherosclerotic plaques tend to develop quietly and are essentially asymptomatic when they remain intact. However, once progress, the ruptured plaques can lead to atherosclerotic thrombosis and a host of attendant complications. Therefore, early diagnosis as well as risk stratification -separating rupture-prone unstable plaques from relative stable plaque- is vitally important (2, 3).

**Figure 1:**
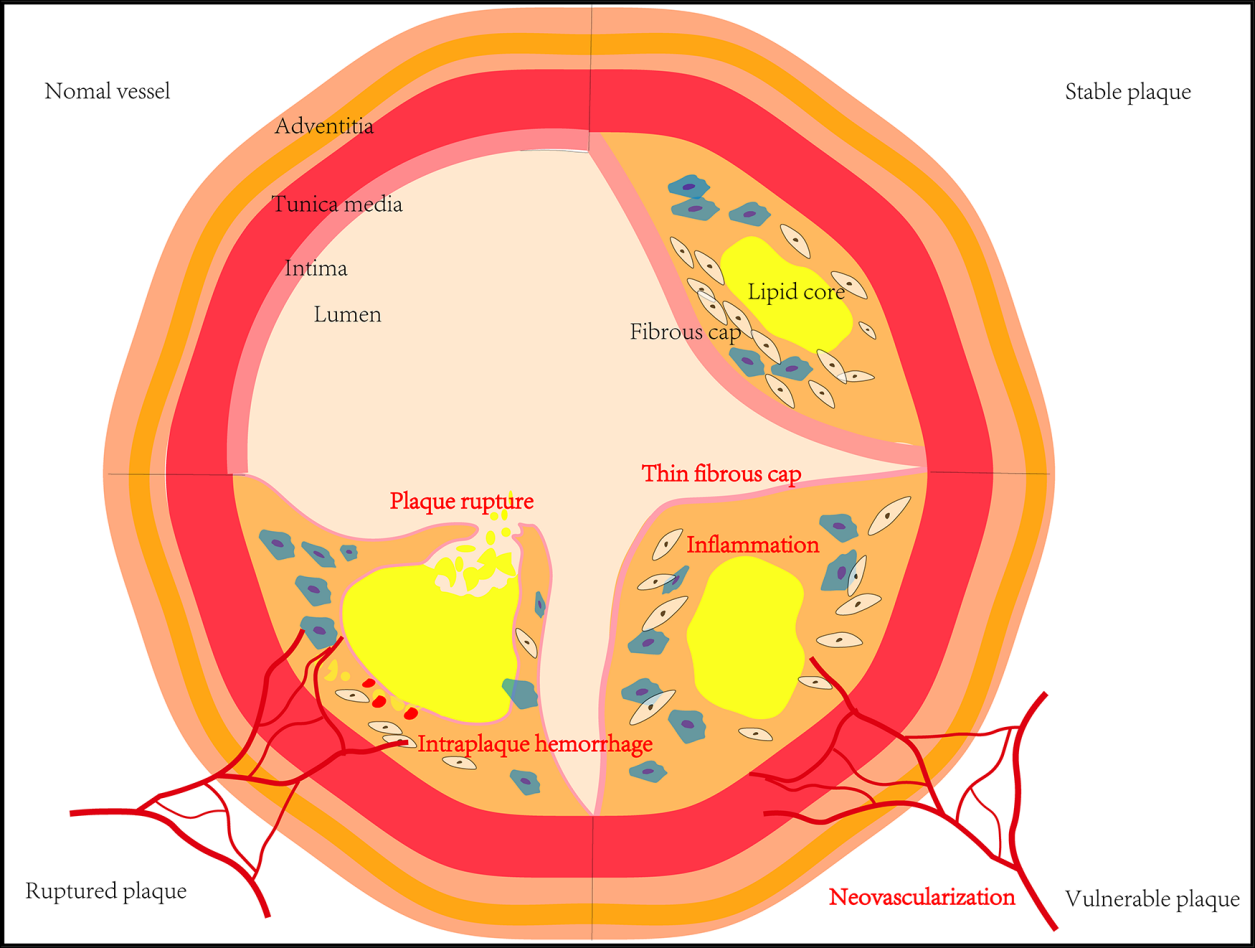
The development and main features of atherosclerotic plaques.

Due to technical and cognitive limitations, previous studies on atherosclerotic plaque have largely been hampered. The size of atherosclerotic plaque and the accompanying luminal narrowing were previously thought to be closely related to acute ischemic cardiovascular events. However, increasing evidence shows that the composition of atherosclerotic plaques is more relevant to acute ischemic cardiovascular events (4). Certain structures and components of atherosclerotic plaques, including intraplaque hemorrhage, large lipid necrotic core, thin fibrous caps, inflammation, and neovascularization, are all been considered promising rupture-prone (5, 6) (Figure 1). In addition, understanding the molecular and cellular mechanisms of atherosclerotic plaque development as well as researching new treatments to prevent or treat acute cardiovascular events are of great significance for both basic research and clinical practice (7).

Several imaging technologies have been developed to assess atherosclerotic disease, among which ultrasound has obvious advantages over other imaging modalities in terms of wide availability and low cost (8). In recent years, with the rapid development of technology, emerging ultrasound techniques including contrast-enhanced ultrasound (CEUS), Doppler ultrasound, ultrasound molecular imaging (UMI), ultrasound elastography and intravascular ultrasound imaging (IVUS) can not only screen and diagnose vulnerable plaques, but also propose the risk classification of vulnerable plaques through different ways, which will be greatly meaningful for the clinical management of patients with atherosclerosis.

Based on the latest literature data, we aim to provide an updated, objective, and comprehensive summary of the current progression of novel ultrasound techniques including the imaging principle, the application and development prospects as well as their limitations.

## 2. Contrast-enhanced ultrasound

CEUS generally makes up for the deficiency of conventional B-mode and color Doppler ultrasound which have long been challenged by their insufficient value to identify components and neovascularization within the plaque (9). It is a rather novel technique with the application of contrast agents in the form of microbubbles (10). In order to circulate freely in the bloodstream like red blood cells through capillaries, these microbubbles are designed into core-shell inflatable microspheres typically smaller than 7 µm in diameter. The shell is usually composed of proteins, lipids, polymers, surfactants, or a mixture, and thus constitutes a barrier between the surrounding environment and the encapsulated gas inside (11). The composition of the shell also determines the hardness of microbubbles, their susceptibility to recognition by the reticuloendothelial system, and their vandalism resistance in high-intensity ultrasonic fields. In addition, the air core encased inside the microbubble considerably enhances the backward scattered acoustic signal (12) (Table 1). Several types of microbubbles such as the Sonovue series are the main ultrasound contrast agents currently approved and recommended for clinical use (13). On the other hand, the conventional diagnostic ultrasound imaging frequency is usually less than 7.5 MHz in clinical practice. However, higher spatial and temporal resolution is needed for the detection of fine vascular structures in preclinical applications, with 15–55 MHZ generally recommended (14). Besides, the penetration depth of CEUS is relatively poor compared with standard B-mode imaging. Although lower imaging frequencies improve penetration, spatial resolution will be negatively impacted. It may be useful for CEUS to use higher-frequency curvilinear transducers and high-frequency linear transducers to improve spatial resolution; however, the microbubble signal may not be as strong, resulting in an overall darker image (15). In theory, these gas-filled microbubbles can stay at the site of the capillary bed, and oscillate upon interacting with the ultrasound wave thus enhancing the reflected ultrasound signal, and improving the visualization of small vascular beds (16).

**Table 1:** The main composition of the microbubble and the function of each part.

Component items	Composition	Function
Shell	Proteins, lipids, polymers, surfactants or a mixture of them	♦ Constitute a barrier ♦ Determine the hardness ♦ Affect the susceptibility of recognition by the reticuloendothelial system ♦ Vandalism resistance
Core	Gas	Enhance the backward scattered acoustic signal
Diameter	1–10 µm	/

As is strongly recommended by the European Federation of Societies for Ultrasound in Medicine and Biology, CEUS has opened a new field of vision for the study of arterial pathology for it can not only quantitatively assess the degree of atherosclerosis stenosis, but also qualitatively assess the vulnerability of plaque based on the presence of ulceration, neovascularization, and inflammatory infiltration (17, 18). It is proved that the extent of intra-plaque neovascularization (IPN) displayed on CEUS has a good correlation with histology. In areas where plaques presented a larger degree of contrast enhancement, the corresponding region on histology also had increased density (19).

**Figure 2:**
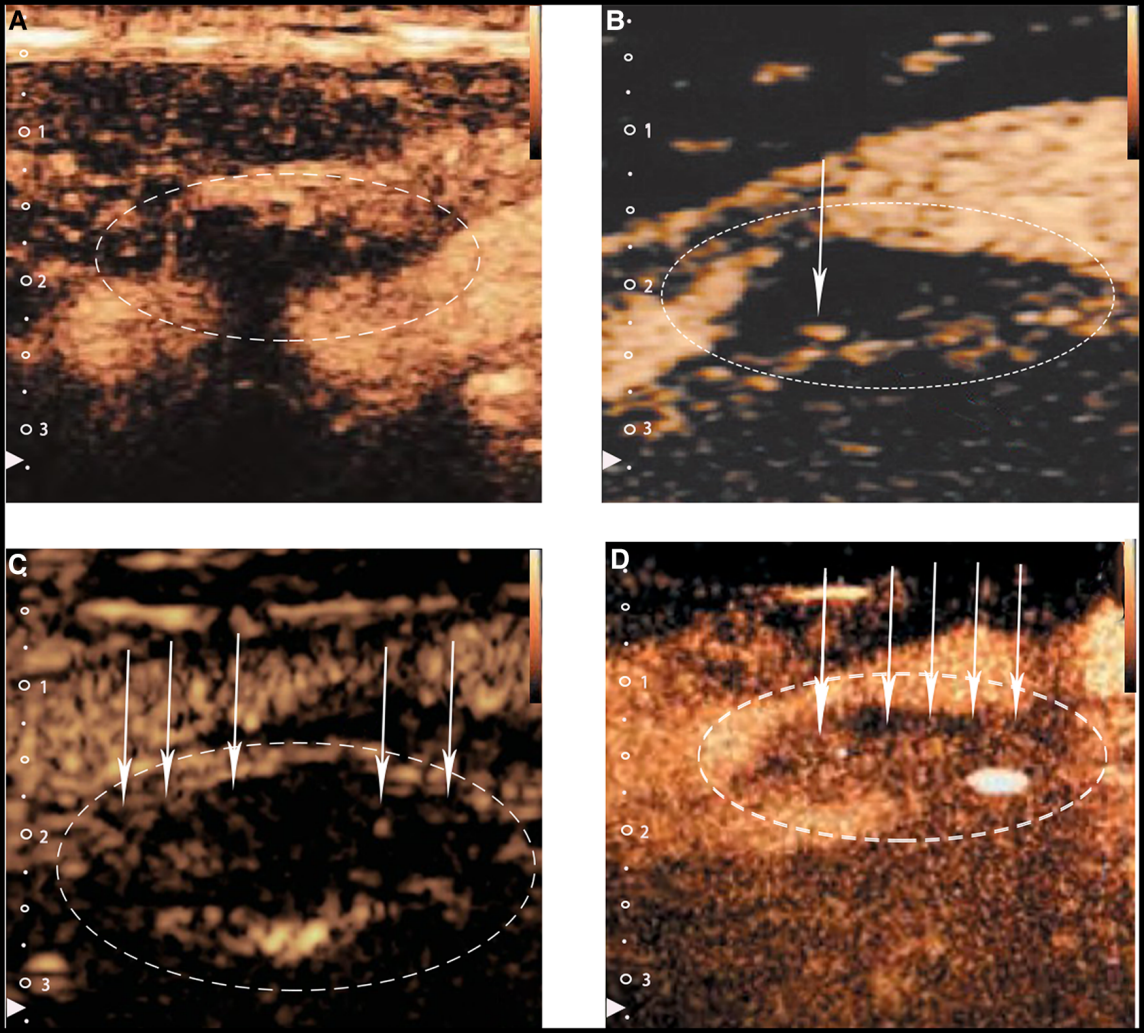
Typical CEUS images of carotid plaques. (Panel **A**) Shows no visible microbubbles in the plaque (grade 0). (Panels **B,C**) Show minimal microbubbles confined to the shoulder or the adventitial side of the plaque (grade 1). (Panel **D**) Shows plentiful microbubbles throughout the plaque (grade 2). The white arrows and dotted lines depict intraplaque contrast microbubbles.

The utility of CEUS in the evaluation of carotid IPN to reclassify patients into more accurate risk categories has been confirmed by several studies. Based on the existence and location of ultrasound microbubbles within each plaque, IPN is typically graded into three levels in CEUS: grade0-no visible microbubbles in the plaque; grade1-minimal microbubbles confined to the shoulder or adventitial side of the plaque; grade2-plentiful microbubbles throughout the plaque (16, 20, 21) (Figure 2). Although mean carotid intima-media thickness (CIMT), maximum plaque height (MPH) and total plaque area (TPA) are typical indicators often used to measure the severity of atherosclerosis in patients, Mantella et al. proved that the carotid IPN score derived from CEUS was even more sensitive than CIMT, MPH or TPA for predicting participants who suffered from serious coronary artery disease (CAD). Furthermore, the study took an IPN score ≥1.25 as a proper cut-off value to predict significant CAD (20), yet Song et al. regarded the IPN score of 2 as the suitable predictor to predict the high rate of stroke recurrence (22). So to date, no consensus on the best predictive IPN score detected by CEUS capable of predicting significant CAD has been reached when taking IPN as an independent predictor of CAD.

As mentioned above, IPN was usually graded by a semiquantitative image analysis relying on the visual assessment which lacks internal standards, nor do IPN suitable for assessing highly calcified plaques, so divergence about the objectivity of CEUS simply employing IPN to evaluate vulnerable plaque still exists (23). Notably, Boswell-Patterson et al. employed a new measurement parameter- neovascularized enhancement ratio (NER) -to analyze complex atherosclerotic plaque models, which allows highly calcified plaques to be analyzed. The formula of NER is (AP × PER) − (AC × CER)/(AP − AC), where Ap equals plaque area, AC equals calcified area, PER equals plaque enhancement rate and CER equals calcified area enhancement rate. NER in this study also shows a positive relationship with IPN volume (24). Recently, Lyu et al. proposed that the direction of contrast agent diffusion may severe as another complementary method for the prediction of unstable atherosclerotic plaques. Apart from the characteristic contrast filling of the depressions at the plaque-lumen boundary, microbubbles spreading from the arterial lumen and into the plaque in the form of rotating moving bright spots or lines can also be a complementary method of detecting plaque rupture (25). Another study reported that incorporating the analysis of stress and strain distribution apart from IPN can also improve the accuracy in the assessment of plaque rupture with neovascularization and IPH (26). Furthermore, the current CEUS-assessed plaque is mainly based on 2-D imaging which is prone to be interfered with by acoustic shadowing (23). The newer three-dimensional CEUS requires more validation studies on its function of further improving visualization and grading the unstable plaques (27). All of these studies pave the way for a more objective measurement of vulnerable plaque and clinical identification of the plaque progression.

To sum up, although it remains challenging to reach a consensus on the diagnostic standards and limited by some intrinsic factors such as artifacts and rather short enhancement time, contrast-enhanced ultrasound has obvious advantages over conventional US in providing quantifiable data and better image quality for identifying Intraplaque neovascularization in rupture-prone vulnerable plaques and formulating a more accurate risk stratification strategy.

## 3. Doppler ultrasound

Doppler ultrasound is a rapidly developing technology to evaluate vulnerable plaques from the perspective of blood flow visualization, among which ultrasonic microflow imaging (SMI) and ultrafast ultrasound imaging (UF) are the typical representatives.

SMI separates the blood flow signal and the overlapping tissue movement artifact by adaptive calculation method to accurately detect the low-velocity blood flow signal, which is useful for detecting neovascularization (28). Grading criteria of SMI for plaque neovascularization (29): Grade 0, no blood flow signal in plaques; Grade I, blood flow signal in the shoulder or base part of plaques; Grade II, diffuse blood flow signals in the plaques.

As we discussed in Part I, contrast-enhanced ultrasound is also valuable for detecting neovascularization, but it is susceptible to interference from intrinsic factors such as artifacts, and its enhancement duration is short. Most worryingly, CEUS requires intravenous contrast, which can cause pain and anxiety in patients (30). By contrast, SMI uses a new adaptive algorithm to identify and eliminate motion artifacts and preserve the smallest low-velocity blood flow signals, thus showing plaque neovascularization in high-resolution detail (31). In addition, there is no need to wait for the contrast agent to be distributed to blood vessels. The time required to observe new blood vessels is much shorter than that of CEUS by just changing the inspection mode to SMI mode by pressing the switch on the ultrasound system (32).

Zhang, H. et al. (33) and Oura, K. et al. (34) both compared the value of ultrasound microflow imaging (SMI) and contrast-enhanced ultrasound (CEUS) in the diagnosis of carotid plaque neovascularization. The results showed that patients with blood flow detected by SMI tended to also show plaque enhancement signals in CEUS with good consistency. Guo, Y. et al. (35) also studied the CEUS and SMI enhancement grade in different thicknesses of plaque plaques that thicker plaques showed a higher density of neovascularization and were more vulnerable. Moreover, studies have shown that SMI can detect more neovessels than contrast-enhanced ultrasound, especially when there is less neovascularization. This may be because SMI allows repeated scanning in many different parts and directions of the plaque (36). The correlation between SMI and histology has also been verified in corresponding studies (36, 37). Therefore, as a simpler and safer technique, SMI provides a new evaluation method for classifying plaques with different echo types. However, at present, SMI technology still has some difficulties in capturing neovascularization with blood flow velocity less than 0.4 cm/s, and there is still a lack of objective quantitative criteria for SMI, which may be the direction of future research.

Ultrafast ultrasound imaging (UF) is capable of capturing images at a frame rate 100 times faster than traditional imaging by using vector Doppler imaging to assess the flow velocities at each point on the image (38, 39). Goudot, G. et al. (40, 41) validated the feasibility of UF in measuring wall shear stress in carotid plaques and established wall shear stress values for different vulnerable carotid plaques. In the course of atherosclerotic plaque development, the advanced plaques begin to invade the lumen, and the endothelial cells begin to experience high shear stress (42). High maximum shear stress is associated with hemorrhage and calcification within the plaque (43). Therefore, accurate measurement of wall shear stress by UF lays a foundation for better characterization of plaques.

In conclusion, from the assessment of plaque microvascularization to calculation of the wall shear stress, the development of new Doppler techniques provide more dimensions and powerful tools for the assessment of atherosclerosis.

## 4. Ultrasound molecular imaging

**Figure 3:**
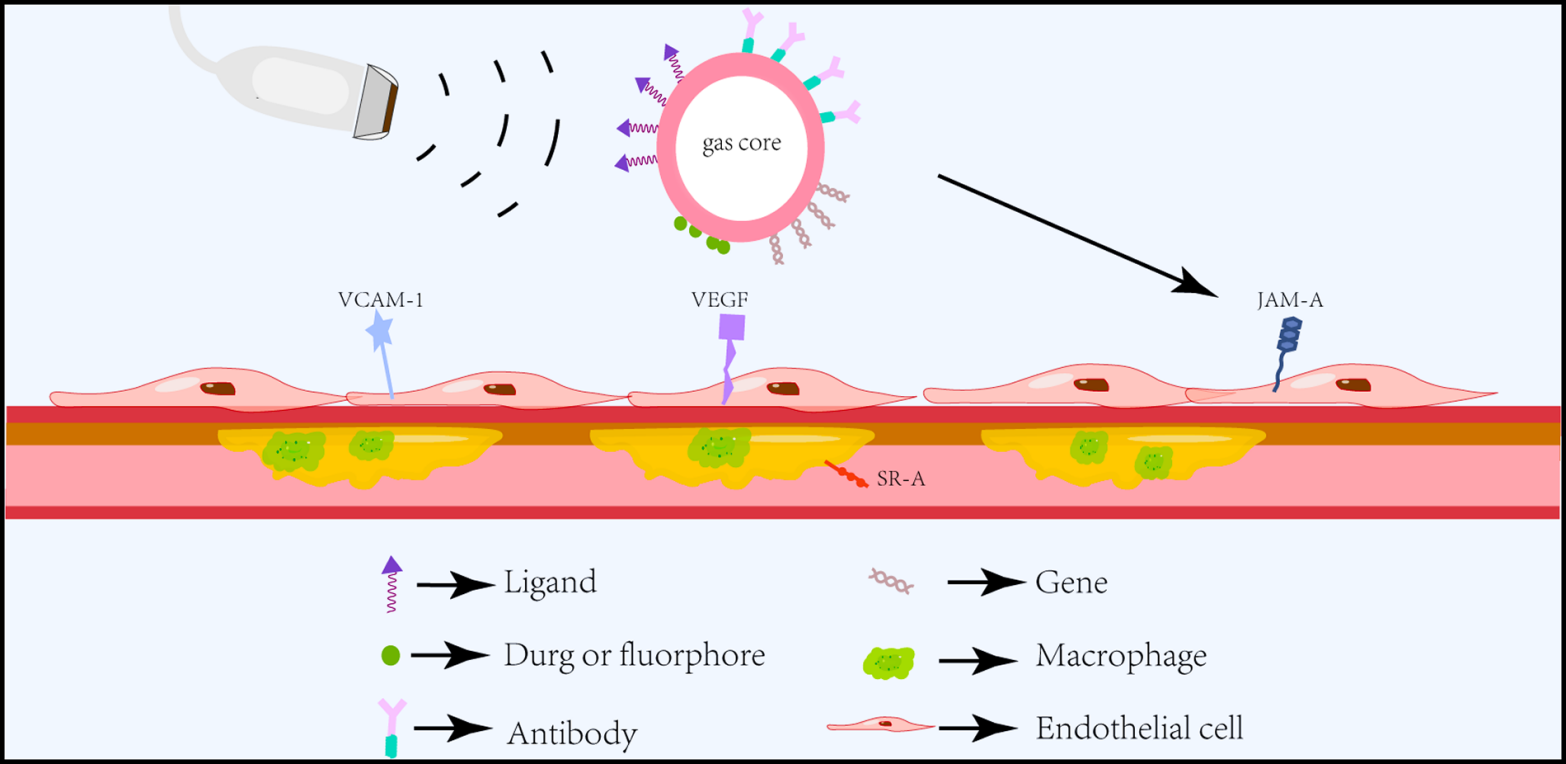
Schematic diagram of ultrasound molecular imaging. Functionalized microbubbles carrying different ligands can specifically and actively target different atherosclerotic endothelial or intraplaque biomarkers under the action of ultrasound and deliver drugs or genes for targeted therapy.

Molecular imaging is an evolving discipline that enables noninvasive visualization, assessment, and quantification of specific biological processes at the cellular level in living subjects (44). These microbubbles possessing specific affinity towards vascular biomarkers of disease can not only improve their accumulation in tissues but also reduce off-target effects, improve safety, and is a promising approach for clinical applications (45) (Figure 3). The functionalization of microbubbles with different targeting ligands to assess atherosclerotic plaques in the early stage and the use of microbubbles for drug and gene delivery has been a hot topic of research in recent years (46, 47).

The development of atherosclerosis is often accompanied by significant changes in inflammatory biomarkers, such as TNF-α, and CRP (48). However, these factors can be elevated in a wide range of pathophysiological activities and are not specific enough to be used in the specific diagnosis of atherosclerosis. Therefore, there is an urgent need to test for biomarkers that are prominent in the atherosclerotic process (Table 2).

**Table 2:** Examples of targeted ultrasound molecular imaging in the identification and treatment of vulnerable plaque (2017–2022).

Shell	Core	Molecular process	Molecular target	Species	Main result	References
Phospholipid	Perfluorinated	Activated platelets	GP Ib and GP IIb/IIIa receptor	Mice	The reactiveness of ultrasound molecular imaging was different within the models according to the type of plaque.	(49)
Phospholipid	Decafluorobutane	Vascular endothelium	VCAM-1	Mice	Signals increased from 8 weeks to 32 weeks age in ApoE-/-mice in different stages.	(50)
Phospholipid	Decafourobutane	Activated platelets	GPIIb/IIIa receptor, selectin ligand	Mice	Dual targeting of microbubbles led to greater capture efficiency, especially at low to intermediate shear stress.	(51)
Phospholipid	Decafluorobutane	Vascular endothelium	P-selectin, VCAM-1, VWF and LOX-1	Mice	The signal for all four targeted microbubbles was significantly higher than that for control microbubbles in DKO mice, and was three to sevenfold higher than in wild-type mice, with the highest signal achieved for VCAM-1 and VWF.	(52)
SonoVue	Sulfur hexafluoride	Smooth muscle cell	miR-21	Mice	Local delivery of a miR-21 mimic rescued the vulnerable plaque rupture phenotype.	(53)
PBCA		Endothelial Dysfunction	JAM-A	Mice	The increment in JAM-A expression and JAM-A-targeted microbubble echogenicity was higher than in controls, peaking after 2 weeks.	(54)
Phospholipid	Perfluoropropane	Leucocytes	VCAM-1, ICAM-1, P-selectin	Mice	The microbubbles had a high affinity to inflammation in both static and dynamic flow conditions. Significantly enhanced ultrasound imaging signals were achieved in detecting the atherosclerosis progress when compared with the single- or dual-targeted microbubbles. Revealing a potential therapeutic efficacy of atorvastatin for early-stage atherosclerosis.	(55)
USphere™Labeler	/	Inflammatory response	IL-8	Rabbits	The peak intensity (PI), microvessel density (MVD), and macrophage count of the pretreatment group were significantly higher than those of both the control and IL-8 groups. Ultrasound-delivered IL-8 monoclonal antibodies alleviate inflammation within atherosclerotic plaques.	(47)
/	Decaflurobutane	Endothelial	VCAM	Mice	Microbubble showed increased attachment under continuous flow with increasing shear stress. UMI in double-knockout mice showed signal enhancement in early and late atherosclerosis in wild-type mice. UMI in human endarterectomy specimens showed a 100% increase in signal.	(56)
Biocompatible lipid	Sulfur hexafluoride	Endothelial	VEGFR-2	Rabbits	Targeted microbubbles exhibit high stability, can effectively identify atherosclerotic plaques, and show outstanding capability for ultrasound molecular imaging.	(57)
SonoVue	Sulfur hexafluoride	Inflammatory responses in macrophage	Glycogen synthase kinase (GSK)-3b	Rabbit	Vulnerable plaque factors and inflammation were suppressed *in vitro* and *in vivo* and changed the cytoskeleton of the foam cells *in vitro*.	(58)
Phospholipid	Decafluorobutane	Anti-inflammatory and anti-thrombotic	VCAM-1, *P*-selectin, VWF, GPIb*α*	Mice	All the remote plaque adverse changes were inhibited by anti-IL-1β therapy.	(59)
/	Perfluoropentane/ Fe3O4	Macrophages/ Activated platelets	SR-A/ (GP) IIb/IIIa	Mice	A high binding Affinity both for activated macrophages and blood clots. Effectively induce macrophage apoptosis, and destroy the thrombus.	(60)
Phospholipid	Perfluoropropane	Activated platelets	GPIIb/IIIa receptors	Mice	Advanced atherosclerotic plaques were rapidly detected and simultaneously giving targeted therapy by dissolving activated and aggregated platelets.	(61)
Phospholipid	Sulfur hexachloride	Endothelial cells	VEGFR-2	Mice	The acquisition of ultrasound information on atherosclerotic plaque neovascularization were enhanced.	(62)
PLT vesicles/ Sono Vue™	RAP@NPs	mTOR signaling pathway	mTOR	Mice	The targeting ability of nanoparticles to atherosclerotic plaques were increased by improving the efficiency of RAP release and the destruction of neovascularization in the plaques.	(63)
Phospholipid	C3F8	Apoptosis of macrophage	Annexin V	Mice	Strong and sustained echo enhancement were shown in plaque area of aortic arch *in vivo*. Imaging sensitive plaques presented more significant pathological changes with several vulnerable plaque features and abundant TUNEL-positive areas.	(64)

First of all, the initial hallmark of atherosclerotic lesion development is endothelial dysfunction and concomitant inflammatory activation, which promote the recruitment of monocytes to the arterial wall, precede the development of plaque and play a role in the occurrence and progression of plaque (65, 66). VCAM-1 and VEGF are classic inflammatory markers when vascular endothelial dysfunction occurs (67, 68). In the past, many studies have taken VCAM-1 and VEGF as targets to develop targeted microbubbles, which have initially realized the detection of early atherosclerosis (56, 57). Further research revealed that one of the main triggers for endothelial dysfunction is altered luminal blood flow, particularly at bifurcations and vessel bends where molecular changes in the endothelium may become apparent (69). Many studies have shown that junctional adhesion molecule A (JAM-A) is one of the most sensitive biomarkers for acute changes in local blood flow and is specifically upregulated on endothelial cells at sites of atherosclerosis predilection (70). As demonstrated by Curaj et al. using antibody-targeted poly microbubbles, transient blood flow changes cause JAM-A rearrangement in the endothelium, and JAM-A-targeted microbubbles can facilitate the early detection of cardiovascular risk areas and play an important role in preventing their progression to irreversible pathology (54).

Besides, macrophages, which account for more than 80% of all cellular components, are extremely abundant in vulnerable plaques and play vital immune and inflammatory roles in both the initiation and progression of vulnerable plaque pathology (71). Macrophages are an important target for diagnostic imaging and new therapies for atherosclerosis. Depending on their polarization status, macrophages function to promote or inhibit atherosclerotic inflammation (72). For instance, researchers have targeted SR-A, which is usually overexpressed on activated macrophages but not unactivated macrophages or other normal vascular wall cells, to distinguish vulnerable plaques (60). This provides a reliable and more specific means of diagnosing vulnerable phenotypes before they are visible morphologically, which substantially facilitates clinicians to set risk evaluation and secondary prevention strategies for the patients at an early stage.

Nonetheless, several existing bottlenecks for ultrasound molecular imaging need to be tackled. The first is its security in clinical applications. Traditionally, the conjunction of targeted ligands to the shell of microbubbles was characterized by a multistep, biotin-avidin-biotin bridging process, which could probably lead to the binding of endogenous biotin (73). Innovatively, Punjabi et al. recently combined microbubble with a nanobody, the smallest possible (10–15 kDa) antibody-derived polypeptide which did not induce complement-triggered cytotoxicity nor bind to Fc receptors on immune and other types of cells. The result indicated a totally increased signal in human endarterectomy specimens compared with the control group (56). So, the use of nanobodies or single-domain antibodies instead of full-size antibody ligands may pave the way for a more secure clinical translation of UMI to detect early atherosclerotic changes.

Secondly, the task to increase the affinity adhesion of targeted microbubbles remains challenging. Microbubbles have the probability to detach from the endothelial target spot, resulting in short contact time and insufficient cell adhesion, particularly in areas where exist high blood shear stresses such as vessel bifurcation (74). Some scholars attempted to utilize external forces such as acoustic radiation force and magnetic field force (75), while other researchers explored multi-targeted microbubbles to promote MB adhesion. Yan et al. developed triple-targeted microbubbles carrying VCAM-1, anti-ICAM-1, and synthetic polymeric sialyl Lewis X (sex) on the surface. Unlike single- or dual-targeted microbubbles, significantly enhancing ultrasound imaging signals were observed in triple-targeted microbubbles even under high shear stress (55).

Furthermore, the usual size of microbubbles of 1–5 µm limits their ability to effectively aggregate in tissue (76). Studies have demonstrated that nano-ultrasound bubbles under 780 nm in size can easily pass through fine capillaries and lymphatic endothelium, thus targeting plaque sites with nano-scale ultrasound contrast agents is expected to better enable early diagnosis of atherosclerosis. As shown in the study of Zhang et al. who designed a nanosized ultrasound contrast agent carrying anti-VEGFR-2 to image the vulnerable plaque in rabbit abdominal aorta, these nano-ultrasound bubbles smaller than the size of the open pores of atherosclerotic plaques significantly extravasate and remain at the plaque site, showing stronger echogenic signal within atherosclerotic plaque and lasted for 1–2 min (57).

Last but not least, more attention should be put on the optimization of the microbubble surface composition and setting a standardization of measurement protocols and quantification methods (10). Still, ultrasound molecular imaging and diagnostics are mostly limited to pre-clinical models and have not been tested in primates or humans up to now, so studies in large models are needed.

## 5. Elastography

Ultrasound elastography can quantify the mechanical characteristics of plaques, and reveal their composition and vulnerability by analyzing tissue displacement in response to either external (focused acoustic radiation) or internal (variation in blood pressure) mechanical excitations (77, 78). Depending on different motivation methods, ultrasound elastography can be classified into two imaging modes: strain elastography (SE) which utilize internal or external stress excitation, and shear-wave elastography (SWE) which utilizes shear wave excited by ultrasound.

### 5.1. Strain elastography

SE measures the plaque displacement gradient under different levels of stress induced by an external force and calculates semi-quantitative parameters such as strain, strain velocities, or strain rate (78). Since fatty elements are much softer than fibrous tissue, plaques with larger local deformations and more complex distribution of strain are more likely to be vulnerable (79). Besides, Liu et al. validated the *in vivo* inter-observer repeatability of ultrasound elastography in identifying vulnerable plaques with an intraclass correlation coefficient of 0.66 between the two operators (80).

Nevertheless, there is no consensus on the optimal imaging parameters up to now. The magnitude of plaque strain such as local maximum, mean, and minimum values or the whole spatially averaged strain are typically been considered suitable quantitative indices for plaque elastic assessment (81). Moreover, Huang et al. put forward that the vulnerability of the carotid atherosclerotic plaques can be better quantified by textural features -contrast, homogeneity, correlation, and angular second moment. In his study, the textural feature achieved 83.8% accuracy in plaque classification while the contrast was 81.3% (82). Another study conducted by Roy Cardinal et al. demonstrated that the ratio of cumulated axial strain to cumulated axial translation could serve as a novel parameter for detecting the vulnerability of plaque (83).

### 5.2. Shear-wave elastography

**Figure 4:**
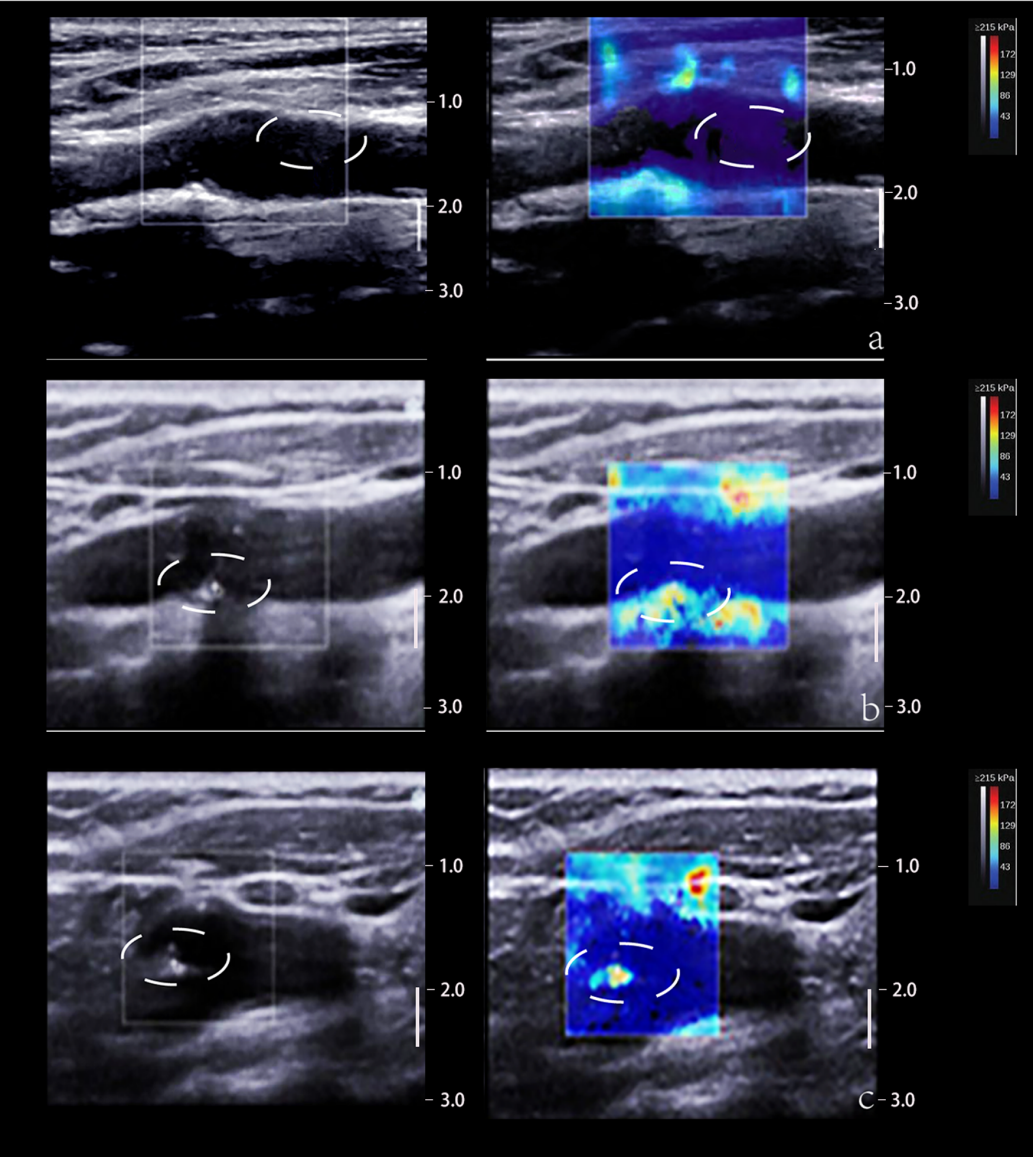
2D and SWE imaging of plaques with different hardness. (Panel **A**) The 2D image displays a predominantly hypoechoic plaque and the SWE image displays a blue color at the plaque, suggesting a soft texture. (Panel **B**) The 2D image shows a predominantly mixed echogenicity of the plaque and the SWE image shows a red-blue mix at the plaque, suggesting that the texture of the plaque is between soft and hard. (Panel **C**) The 2D image demonstrates that the plaque is predominantly strong echogenic and the SWE image demonstrates a red color at the plaque, suggesting a hard texture. The white dotted lines depict the plaque area.

SWE estimates tissue elasticity by tracking the transverse velocity of the tissue after it has been subjected to an external force. The transducer emits a transverse wave through an acoustic radiation force pulse (ARFI) and measures the speed of the transverse wave propagation through the tissue, expressed as Young's modulus (YM), which reflects the tissue's resistance to elastic deformation and is largely depended on the composition of the tissue. Soft tissues such as lipid cores in plaques tend to show obvious elastic deformation, lower YM and lower transverse wave velocity; whereas harder tissues and lesions show less elastic deformation (77, 84) (Figure 4).

The potential clinical value of SWE in assessing the elasticity of carotid atherosclerotic plaques has been demonstrated in both vitro and vivo studies, and is demonstrated particularly useful in distinguishing asymptomatic plaques from symptomatic plaques (85). In the study of Skoloudik et al., they divided asymptomatic plaques into stable and progressive groups (86). The results of this study clearly showed that there were significant differences between stable and progressive plaques in terms of measured mean, minimum, and maximum Young's modulus. Young's modulus measured in asymptomatic progressive plaques was even lower compared to symptomatic plaques, although there was no significant difference. Therefore, SWE measurements of plaque elasticity can be used as an adjunct parameter for the indication of carotid endarterectomy or angioplasty and stenting when asymptomatic carotid stenosis of >50% is first detected.

However, the complex composition of atherosclerotic plaques strongly requires a combination of other parameters to distinguish. In vitro studies have shown that a combination of spatio-temporal and frequency-dependent shear wave analysis can be used to non-invasively assess the characteristics of atherosclerotic plaques *in vivo* (87). Recently, Marlevi et al. further showed in an *in vivo* study that vulnerable plaques have significant group velocities and frequency-dependent phase velocities compared to other types of plaques. More interestingly, this parameter was also used in the study to classify specific components within the plaque, including thin fibrous caps, lipid-rich necrotic cores, and intraplaque hemorrhages (88). In addition, Torres et al. combine signal correlation and signal-to-noise ratio (SNR), expressed as a decimal logarithm of the acceleration variance, either directly or through displacement variance, called “log(Vo A)” to successfully describe the composition and structure of human carotid atherosclerotic plaque (89). Overall, these results are all clinically useful for predicting stroke risk and facilitating medical management.

As such, elastography provides a more in-depth assessment of plaques by bringing a novel dimension-analyzing tissue displacement- to clinical use. Still, it remains a challenge to reach intra/inter-observer reproducibility and agreement on standardized cutoff value (Table 3).

**Table 3:** Applications of ultrasound elastography in the identification of vulnerable plaque (2017–2022).

Patients involved	Plaques	Method	Measurement parameters	Reference standard	Main conclusion	References
52	80	ARFI	Texture analysis(strain rate images contrast, homogeneity, correlation and angular second moment)	MRI	Plaque classification using texture analysis is feasible. Larger local deformations and higher complexity of deformation patterns are more suggestive of plaque vulnerability.	(82)
25	26	ARFI	Peak displacement	Histology	ARFI can distinguish soft from stiff atherosclerotic plaque components and delineating fibrous cap thickness.	(90)
31	31	SWE	Maximum axial strain, cumulated axial strain, mean shear strain, cumulated shear Strain, cumulated axial translation, cumulated lateral translations, the ratio of cumulated axial strain	MRI	Cumulative axial translation and the ratio of cumulative axial strain to cumulative axial Translation are sensitive parameters for distinguishing between vulnerable and non-vulnerable carotid plaques.	(83)
61	271	SWE	Young's modulus	Neurological symptoms	Young's modulus can be used as an additional method to detect symptomatic carotid plaque.	(85)
/	24	SWE	Plaque geometry, push location, imaging plane, and wave speed metric	Vitro setup	Differentiation of simulated plaques with different mechanical stiffness can be achieved using SWE.	(87)
66	132	SWE	Plaque translation and elastography and echogenicity features	Neurological symptoms	The combination of elastography and echo analysis helps to differentiate plaque in symptomatic patients from asymptomatic ones.	(91)
142	129	SWE	Maximum, minimum, and mean values of shear wave velocity	Neurological symptoms	Echogenic plaques had higher shear wave velocity than echolucent ones.	(92)
25	25	ARFI	Decadic logarithm of the variance of acceleration[log(VoA)]	Histology	Log(VoA) is able to characterize the composition and structure of human carotid atherosclerotic plaques *in vivo* better than PD.	(89)
32	53	ARFI	The maximum 99th percentile of absolute axial strain rate	MRI	Ultrasound-based carotid elastography is reproducible and reliable in differentiating between vulnerable and stable plaques between two operators.	(80)
20	27	SWE	Group velocity and frequency-dependent phase velocities	MRI	The combined group velocity and frequency-dependent phase velocity can improve the ability of SWE to detect vulnerable carotid plaques, providing additional information for assessing plaque characteristics.	(88)
97	97	SWE	Mean, maximal and minimal elasticity	Neurological symptoms	SWE is a promising way to differentiate symptomatic, asymptomatic progressive, and asymptomatic stable carotid plaques.	(86)
46	46	SWE	Stiffness distribution	Histology	In vulnerable plaques, there was a significantly increased percentage of stiffness range of 3–5 m/s.	(41)

## 6. Intravascular ultrasound imaging

Using a miniature ultrasound probe guided to the target site via a trans-vascular guide wire, Intravascular ultrasound imaging contributes to a direct way to visualize the nature of selected atherosclerotic lesions (93). Although Coronary angiography (CAG) has long been recognized as the gold reference standard for the evaluation of coronary artery disease, it is incapable to evaluate the structural composition of unstable plaque and lacks objectivity since it relies heavily on contrast agent-filled vascular contour to assess the diameter of the lumen. IVUS is complementary to CAG in that it visualizes features of atherosclerotic plaques qualitatively within the vessel either from a longitudinal plane or an axial plane (94, 95).

### 6.1. Grayscale IVUS

**Figure 5:**
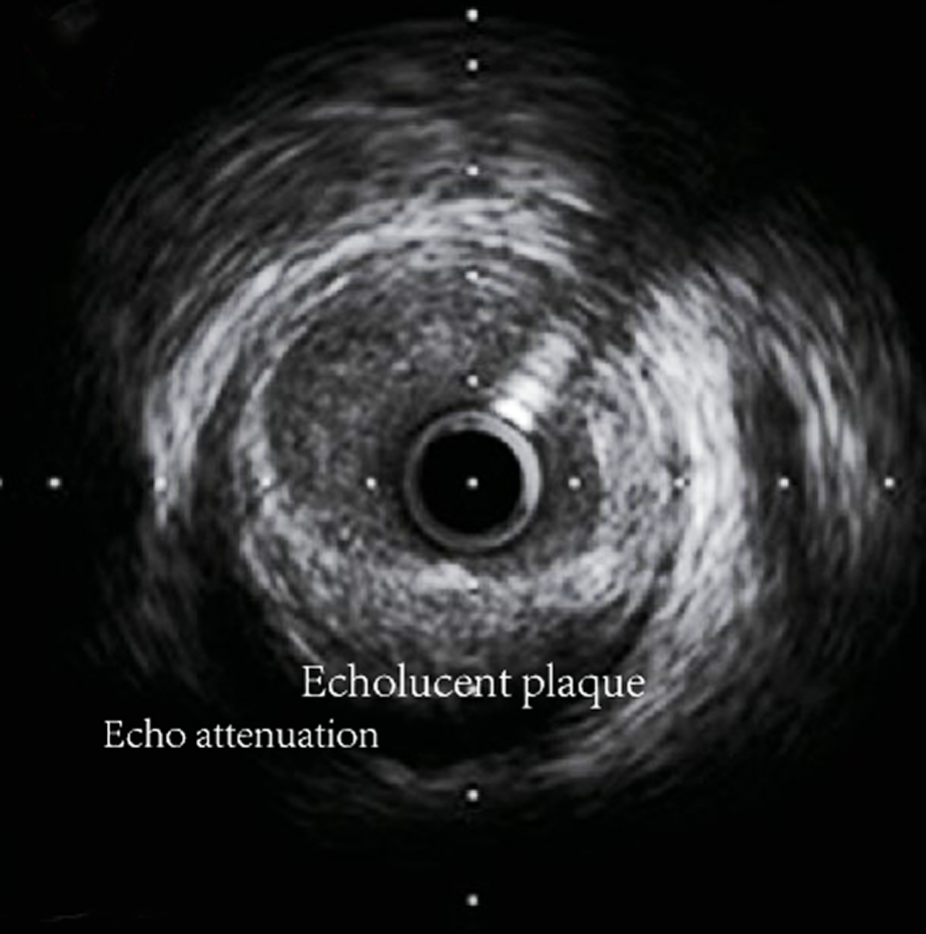
Typical grayscale display of arterial cross-section image obtained by conventional grayscale IVUS.

Conventional grayscale IVUS offers grayscale cross-sections of coronary arteries with an axial resolution of approximately 100–250 µm (96). It determines the characteristics of plaques through the echo intensity of plaques into the following four types: (1) soft plaques: echo lower than the surrounding outer membrane; (2) calcified plaque: echo higher than the surrounding outer membrane; (3) fibrous plaques: moderate echo; (4) mixed plaques: two or more echo signals (97). The attenuated plaques (AP), characterized by hypoechoic areas of deep echogenic attenuation and echogenic hyaline plaques despite the absence of bright calcium and echolucent plaque (ELP), characterized by areas of non-echoic or hypoechoic plaques displayed on the IVUS were demonstrated to be linked with vulnerable plaque phenotype (98). This is mainly due to the fact that plaque hypoechoic areas usually represent plaques that are high in lipids and poor in collagen. Echo attenuation in ultrasound plaques is closely associated with microcalcifications and cholesterol crystals within the lipid-rich necrotic core, which promotes signal reflection and dispersion (99) (Figure 5).

An obvious drawback of greyscale IVUS is its low resolution, which limits detailed visualization of the plaque phenotype. Besides, caused by the strong reflection of intracavitary calcium to ultrasound, it has limited value to assess the calcification (97, 100).

### 6.2. Contrast-enhanced IVUS

As previously mentioned, new microvessels form within the outer membrane of the arterial wall, and the atherosclerotic plaque (i.e., vasa vasorum, VV) is an important marker of plaque inflammation (5, 6), which is a precursor or concomitant factor related to plaque rupture and plaque instability (101). The combination of conventional IVUS with contrast agents, e.g., microbubbles (contrast-enhanced IVUS, CE-IVUS) has proven useful for coronary plaque perfusion imaging and assessment of the amount and distribution of new vessels in atherosclerotic lesions, providing a step forward in the identification of vulnerable plaques (102, 103). An earlier study conducted by Carlier S showed the feasibility of contrast-enhanced IVUS plaque imaging with intracoronary microbubbles (104). But due to limitations at the time, they did not correlate their study with histopathological evidence of vasa vasorum. This was confirmed in a subsequent study by Vavuranakis M et al., who used CE-IVUS to detect and quantify rabbit aortic wall neovascularization (105). The density of VV also shows a positive correlation with plaque progression, inhibiting VV may be a promising approach to delay or even reverse the progression of atherosclerosis (101).

However, detecting VV is challenging because of the low acoustic scattering of blood compared to tissue, making it cumbersome to differentiate between contrast and tissue (usually called contrast-to-tissue ratio, CTR) (106). A new contrast imaging method based on the detection of higher harmonics named Super harmonic (SH) has been proposed, which performed with a low frequency transmitter and high frequency receiver. By increasing the contrast agent signal and suppressing the signal from the tissue, the technique has shown even higher CTR than second harmonic (107, 108). Ma, J. et al. designed a small aperture (0.6 × 3 mm) IVUS probe with dual frequency (6.5 MHz/30 MHz) transducer for high frequency contrast imaging. The microbubble is excited at low frequencies (near resonance) and its wideband harmonics are detected at high frequencies, minimizing the detected tissue backscattering (109). More recently, Lee, J. et al. reported a dual-element focused IVUS transducer, consisting of a 35 MHz ultrasonic transmission element and a 105 MHz third harmonic receiving element, which produces third harmonic images with higher spatial resolution and deeper imaging depth than fundamental wave images (110). In the future, we look forward to additional *in vitro* and *in vivo* trials of atherosclerotic plaque lesions to further validate the clinical value of these IVUS sensors.

### 6.3. Post-processing IVUS

**Figure 6:**
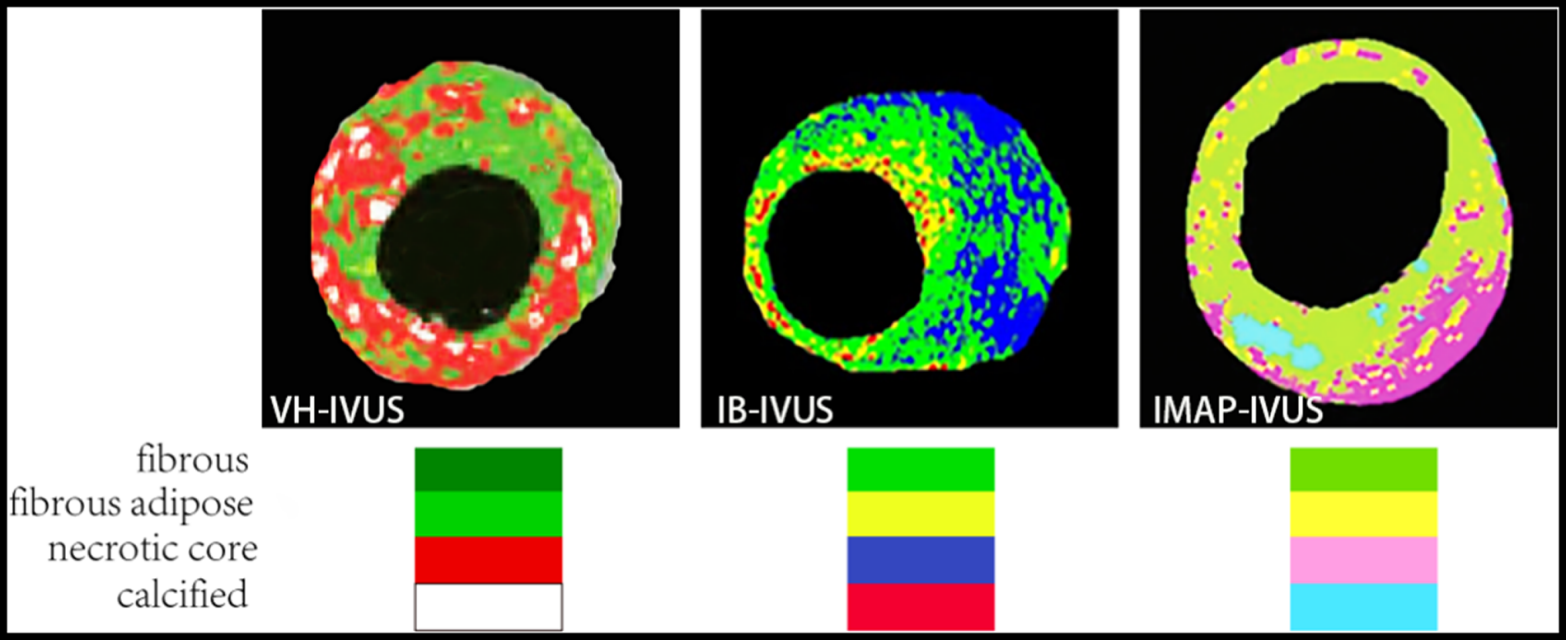
An illustration of color coding methods of VH-IVUS, IB-IVUS, and IMAP-IVUS.

Virtual histology (VH-IVUS), integrated backscatter (IB-IVUS), and IMAP-IVUS are all examples of the products of the evolution of post-processing algorithms. Based on different post-processing algorithms, a comparison of color coding methods of them is presented in Figure 6 (111–113).

Despite different plaque characterization algorithms, the utility of post-processing IVUS in elucidating factors associated with compositional changes suggestive of increased plaque vulnerability is gaining more and more attention. Since current research on premature coronary artery disease is usually focused on genetics and epidemiology, and studies related to coronary plaque characteristics are still scarce, Xie J et al. analyzed the morphological, histological, and phenotypic characteristics of atherosclerotic plaques in 47 patients with premature CAD by iMap-IVUS and compared the differences with 155 patients with later CAD. The results revealed more fibrosis and less necrotic and calcified components within the plaques of patients with premature CAD compared with later CAD (114). This may provide important information for subsequent insights into the development of premature coronary artery disease.

In addition, studies have shown that acute cardiovascular events are usually caused by a vulnerable coronary plaque containing a large lipid-rich necrotic core covered by a thin inflamed fibrous cap, defined as thin-cap fibroatheroma (TCFA) (115). The advent of IVUS holds promise for the study of the impact of tissue characteristics of culprit lesions on myocardial tissue perfusion levels. Several clinical trials have already validated its usefulness in identifying clinical and lesion-related factors that put patients at risk of adverse cardiac events, as well as predicting future acute cardiovascular events.

PROSPECT is one of the most classic and largest of these observational studies. It is a prospective study with a median follow-up of up to 3.4 years and included a total of 697 patients with acute coronary syndromes, each of whom underwent coronary angiography, Grayscale IVUS, and VH-IVUS following percutaneous coronary intervention. Results showed that recurrent major adverse cardiovascular events were associated with both initially treated (culprit) lesions and untreated (non-culprit) lesions. Of the 157 recurrent events where the lesion could be located, 74 (47%) were associated with the original nonculprit lesion. And of the 51 recurrent events associated with non-culprit lesions, 26 (51%) occurred at sites of thin fibrous cap fibroatheroma (TCFA) detected on the basis of VH-IVUS, most of which showed no evidence of severe stenosis on conventional angiography. It is concluded that small lumen area, large plaque burden, and the presence of thin cap fibrous atherosclerosis were three important predictive features of recurrent events (116). This study provides prospective, systematic data on the origin of recurrent ischaemic events by using VH-IVUS to characterize the morphological features of plaques, enabling the prospective identification of thin-cap fibrous atherosclerosis. This also further demonstrates that the development of acute coronary syndromes is not necessarily dependent on the degree of angiographic stenosis at the site and that the development of VH-IVUS has made it possible to characterize the vessel wall using imaging techniques that can be comparable to histological findings.

An EARLY-MYO-ACS prospective observational study conducted on 408 patients investigated the relationship between culprit plaque characteristics before percutaneous coronary intervention (PCI) and myocardial tissue level perfusion after PCI in patients with non-ST-segment elevation acute coronary syndrome (NSTE-ACS) by IMAP-IVUS. The results showed that in these NSTE-ACS patients, an increase in the necrotic portion of the culprit lesion was independently associated with myocardial tissue level perfusion impairment (117). This reveals a valuable application of plaque composition assessment by pre-PCI IMAP-IVUS to predict myocardial tissue level perfusion injury after PCI in patients with NSTE-ACS.

IVUS also gains well recognization and validation for its ability to study mechanisms underlying certain therapies with its high imaging resolution (118). The SATURN trial and IBIS-4 study are classic studies that investigated the effect of atorvastatin and rosuvastatin in CAD and STEMI patients respectively by VH-IVUS-derived plaque components (119, 120). In a vivo study conducted by Andrews J et al., they measured plaque calcification and atheroma volume with IVUS and found that warfarin was independently associated with intravascular calcification in patients with coronary atherosclerosis, but not with atherosclerotic volume, statin therapy, or renal function (121). More recently, in a study combined with 9 randomized clinical trials, the authors first demonstrated a statistically significant independent association between oral calcium and progressive coronary calcification, as assessed by IVUS in terms of plaque calcification and atherosclerotic volume (122). Thus, imaging identification of high-risk patients using IVUS may one day help to justify the focal treatment of vulnerable plaques, and its safety and efficacy will need to be demonstrated in additional randomized trials.

However, in a 4.7-year study of AtheroRemo-IVUS, researchers reported that the small luminal area (minimal luminal area ≤4.0 mm^2^) and large plaque burden (≥70% burden) measured by conventional IVUS, rather than the post-processing-IVUS-derived plaque composition characteristics (e.g., TCFA) by themselves, could predict adverse cardiovascular outcomes. However, no increased risk associated with minimal lumen area was observed in this study group during the previous 1-year follow-up. But the prognostic value of a plaque burden ≥70% was similarly confirmed, although there was inconsistent statistical significance across all the different cardiovascular event endpoints. More interestingly, the independent association between TCFA lesions as a feature of independent vulnerable plaques and 1-year adverse cardiovascular events did not persist at subsequent long-term follow-up. The study suggests possible reasons for this, one important being the dynamic development of TCFA lesions over time, particularly at the proximal end where the plaque burden is greater, where lesions heal more slowly and have a greater propensity to rupture, and therefore this should be taken into account as an important guideline for future clinical trials (123). In addition, as Stone, Get al. noted, these studies only imaged a single segment, and even in the PROSPECT study, only 3 epicardial coronary arteries proximal to 6 cm–8 cm were imaged, so the relationship between the imaged lesion and subsequent acute cardiovascular events using IVUS appears to require further investigation, as most events will originate from non-imaged lesions (124). Moreover, the GLAGOV study also challenged the use of VH-IVUS to assess plaque morphology, as they argued that in the evolocumab trial, VH imaging did not provide any incremental information other than the assessment of changes in plaque burden (125). Thus, it appears that the role of IVUS in quantitatively assessing the relationship between the vulnerable components and the pathogenesis of ischaemic events and the efficacy of various treatments requires further investigation.

## 7. Conclusion and prospect

Vulnerable plaques account largely for the occurrence of serious acute clinical complications. In this review, we made a comprehensive overview of recent updates concerning novel ultrasound techniques including CEUS, UMI, Doppler ultrasound, elastography and IVUS in the identification of vulnerable plaques, with the expectation to offer a more reliable classification of the vulnerable plaque and facilitate clinical risk stratification of the individuals.

Each ultrasound imaging method has its irreplaceable advantages. Contrast-enhanced ultrasound and Doppler ultrasound provide significantly detailed information on intra-plaque neovascularization in plaques which are highly associated with serious coronary artery disease. Ultrasound molecular imaging also gains increasing recognition for its ability to image the target plaque at the cellular or molecular level as well as enhance transfection efficiency to stabilize the plaque. Furthermore, elastography allows for better measurement of vulnerable plaque through analyzing tissue displacement in response to either external or internal mechanical excitations. Last but not least, rising evidence suggests that IVUS is a promising invasive tool for plaque vulnerability assessment which visualizes the nature of selected atherosclerotic lesions directly.

With the progress of various ultrasound imaging techniques, the image quality and measurement dimensions have been greatly improving, but the major challenge remains to reach a consensus on accurate diagnosis and stabilizing the vulnerable plaque. At the same time, the composition of plaques is so complex that evaluating just one plaque component may not be sufficient for risk stratification. As such, a multimodal image that combines the advantage of each imaging mode is a promising development direction, such as CE-IVUS, or IVUS-SE. Vavuranakis, M. et al. used computational analysis of CE-IVUS images to detect and quantify VV in rabbits, which is consistent with histological data (105). PROSPECT II is one of the prime examples and is the first multimodal multicentre study to combine near-infrared spectroscopy and intravascular ultrasound (126). This study greatly extends the value of imaging in the detection of vulnerable plaques before they cause acute cardiovascular events, laying the groundwork for future randomized trials of systemic and focal treatments.

Although is still in the early stage of research, we believe that through the joint efforts of researchers, novel ultrasound techniques will provide patients with more accurate and effective identification of vulnerable plaques. Overall, we provide an overview of the mechanism, advantages, limitations and recent progression of new ultrasound methods in the identification of vulnerable plaques in Table 4.

**Table 4:** An overview of the mechanism, advantages, limitations and recent progression of novel ultrasound methods in the identification of vulnerable plaques.

Novel ultrasound method	Mechanism	Advantages	Limitations	Recent progressions
CEUS	Inject contrast agent microbubbles intravenously to enhance blood flow signals	♦ improve the visualization of small vascular beds ♦ have a good correlation with histolog ♦ promote cardiovascular risk stratification	♦ lack internal standards ♦ be interfered by artifact ♦ limited enhancement time ♦ requires rich experiences in operation	♦ predict significant coronary artery disease and future adverse cardiovascular events (16, 20, 22). ♦ early identification of carotid atherosclerosis (23) ♦ NER, the direction of contrast agent diffusion and analysis of stress and strain distribution may severe as complementary methods for the prediction (24–26)
Doppler Ultrasound	Visualize blood flow and assess the flow velocities	♦ detecting neovascularization ♦ high-resolution ♦ time-saving and safe	♦ can not detect blood flow velocity less than 0.4 cm/s ♦ lack of objective quantitative criteria ♦ low reproducibility	♦ show great consistency with CEUS and histology (33, 34, 36, 37) ♦ measuring wall shear stress (40, 41)
UMI	Gather microbubbles bounded with targeting ligands or antibodies in targeted tissue or organ	♦ targeted imaging at the cellular or molecular level ♦ diagnose vulnerable atherogenic phenotypes before they are morphologically visible ♦ improve transfection efficiency	♦ controversy over its safety ♦ poor affinity adhesion ♦ no standardization of measurement protocols	♦ visualize acute endothelial activation and dysfunction (54) ♦ detect vascular inflammation (56, 57). ♦ alleviate inflammation progression by delivering miRNA or antibodies (47, 53, 58) ♦ multi-targeted MBs (55)
Elastography	Analyze tissue displacement in response to either external or internal mechanical excitations	♦ assess mechanical characteristics and stiffness distribution of plaques ♦ classify fibrous cap, necrotic core, intraplaque hemorrhage	♦ no consensus on theoptimal imaging parameters ♦ Inter-observer and intra-observer variation	♦ validate the *in vivo* inter-obseXSSWrver repeatability (80) ♦ quantify vulnerable plaques by textural features (82) ♦ add the ratio of cumulated axial strain to cumulated axial translation as the novel parameter (83) ♦ VOA may be more consistent with histological thickness than PD (89)
IVUS	Employ a miniature ultrasound probe guided to the target site	♦ visualize the nature of selected atherosclerotic lesions directly ♦ complementary to CAG ♦ multiple planes imaging ♦ evaluate the mechanism underlying certain therapies	♦ invasive ♦ low resolution ♦ imited value to assess the calcification	♦ attenuated plaque (AP) and echolucent plaque (ELP) are highly associated with the prevalence of major cardiovascular events (98) ♦ premature CAD had more fibrotic with less necrotic and calcified components within the plaque than later CAD (114) ♦ increased percentage of necrotic plaque fraction is independently associated with impaired myocardial perfusion (117) ♦ explore several indicators of future severity cardiovascular events (127, 128) ♦ (PSS) and (WSS) may influence plaque development (129) ♦ the efficacy of treatment suppression on atherosclerotic plaques (122, 130, 131)
